# Flexible Multielectrode Array for Skeletal Muscle Conditioning, Acetylcholine Receptor Stabilization and Epimysial Recording After Critical Peripheral Nerve Injury

**DOI:** 10.7150/thno.35436

**Published:** 2019-09-21

**Authors:** Malia McAvoy, Jonathan K. Tsosie, Keval N. Vyas, Omar F. Khan, Kaitlyn Sadtler, Robert Langer, Daniel G. Anderson

**Affiliations:** 1 David H. Koch Institute for Integrative Cancer Research, Massachusetts Institute of Technology, Cambridge, Massachusetts 02139, USA; 2 Harvard/MIT Health Sciences and Technology, Harvard Medical School, Boston, Massachusetts 02115, USA; 3 Department of Biomedical Engineering, Columbia University, 351 Engineering Terrance Mudd Building, 500 West 120th Street, New York, NY, 10027, USA; 4 Department of Chemical Engineering, Massachusetts Institute of Technology, Cambridge, Massachusetts 02139, USA; 5 Institute for Medical Engineering and Science, Massachusetts Institute of Technology, 77 Massachusetts Avenue, Cambridge, Massachusetts 02139, USA.

**Keywords:** flexible multielectrode array, functional electrical stimulation, electromyography, neuromuscular junction, neural interface

## Abstract

Complete re-innervation after a traumatic injury severing a muscle's peripheral nerve may take years. During this time, the denervated muscle atrophies and loses acetylcholine receptors, a vital component of the neuromuscular junction, limiting functional recovery. One common clinical treatment for atrophy is electrical stimulation; however, epimysial electrodes currently used are bulky and often fail due to an excessive inflammatory response. Additionally, there remains a need for a device providing *in vivo* monitoring of neuromuscular regeneration and the maintenance of acetylcholine receptors. Here, an implantable, flexible microelectrode array (MEA) was developed that provides surface neuromuscular stimulation and recording during long-term denervation.

**Methods:** The MEA uses a flexible polyimide elastomer and an array of gold-based microelectrodes featuring Peano curve motifs, which together maintain electrode flexibility. The devices were implanted along the denervated gastrocnemius muscles of 5 rats. These rats underwent therapeutic stimulation using the MEA daily beginning on post-operative day 2. Another 5 rats underwent tibial nerve resection without implantation of MEA. Tissues were harvested on post-operative day 14 and evaluated for quantification of acetylcholine receptors and muscle fiber area using immunofluorescence and histological staining.

**Results:** The Young's modulus was 1.67 GPa, which is comparable to native tendon and muscle. The devices successfully recorded electromyogram data when implanted in rats. When compared to untreated denervated muscles, MEA therapy attenuated atrophy by maintaining larger muscle fiber cross-sectional areas (p < 0.05). Furthermore, the acetylcholine receptor areas were markedly larger with MEA treatment (p < 0.05).

**Conclusions:** This proof-of-concept work successfully demonstrates the ability to combine conformability, tensile strength-enhancing metal micropatterning, electrical stimulation and recording into a functional implant for both epimysial stimulation and recording.

## Introduction

The advancement of restorative bionics has been hindered by the rigidity of silicon, the fundamental platform of all electronics since its implementation in the first transistor in 1947^1^. The mechanical mismatch between the stiff electrode and soft tissue can cause aggregative inflammation at the implantation site^2^. The resulting fibrous tissue may isolate the device from the underlying tissue, limiting the effective transfer of current in both recording and stimulating. A desirable electrode would comprise a flexible material offering low modulus responses to stretching resulting in more elastic deformations and an intimate contact between the moving tissue and the electrode. Flexible electronics have been developed for electronic skins^3^, electronic textiles^4^ and flexible solar cells^5^. Such products consist of metal films deposited on a polymer substrate. The Young's moduli of silicon^6^, polyimide (PI)^7^, SU-8^8^, parylene C^9^ and polydimethylsiloxane (PDMS)^10^ are approximately 25.5 GPa, 8.45 GPa, 5.6 GPa, 4.0 GPa and 1.0 MPa, respectively. Thus, electrodes with polymer substrates cause less tissue damage compared with silicon-based electrodes. PI was chosen as a substrate because of its versatile shape and its relatively mild foreign body response^11^, both of which are useful for removable, non-permanent implants. This material has been widely used for neural surface interfacing^12^-^15^. Xue et al.^16^ designed C-shaped cuff electrodes with gold carbon nanotubes fabricated on a PI substrate about 12 μm in thickness to create a nerve-electrode interface with minimal contact to decrease neural damage.

**Figure 1:**
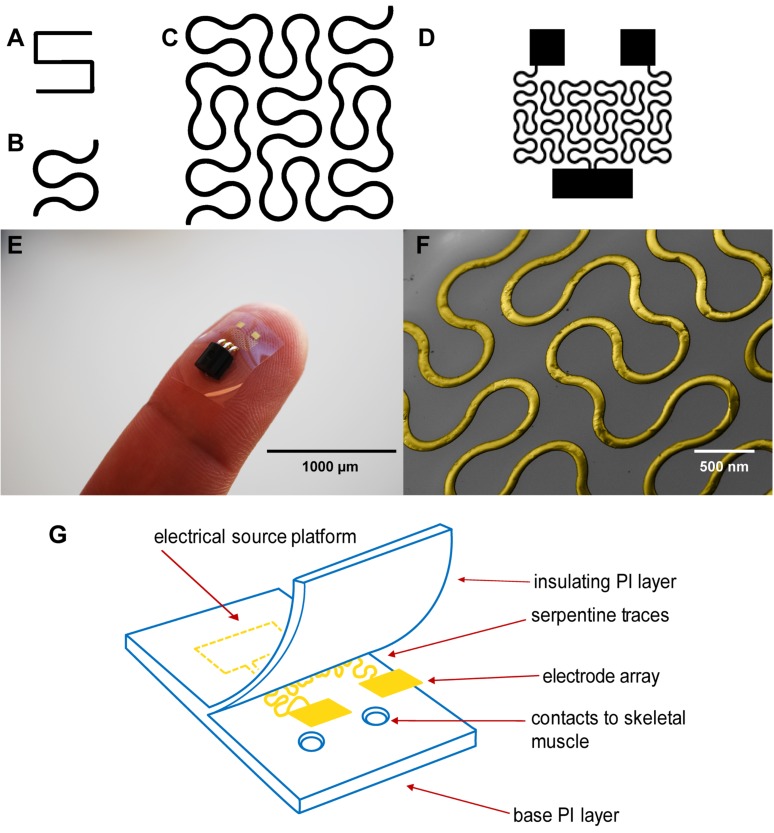
(A) A basic element of a fractal pattern. (B) The hard edges were replaced by arcs for contour. (C) Peano curve variation shown to provide hard-metal traces with tensile strength. (D) The electrode array Peano curve. (E) The flexible microelectrode array (MEA), including a source platform that is 1cm by 1cm in size. (F) Scanning electron microscope image of the gold Peano curve electrode. (G) Schematic of the device components.

Although these PI electrodes are flexible, one limitation is that metal components that serve as the conductive layer embedded within the polyimide substrate including gold, stainless steel, platinum, titanium and iridium have a stiffening effect^17^. In a recent study, hard metal wiring was patterned in fractal motifs (**Figure 1A** and **1B**) and bonded to elastomers to increase stretchability^18^. The Peano curve is a fractal curve that is continuous and space filling^19^. The design can be used in microelectronics for wiring and provides multiaxial support^18^. This Peano motif variation, shown in **Figure 1C**, was utilized for the wiring of our device that connects the contact pads to the source platforms (**Figure 1D)**.

The implantable, microelectrode array (MEA) has been developed to electrically stimulate muscle after peripheral nerve injury in which the nerves to the skeletal muscle have been severed and undergo Wallerian degeneration^20^. Sectioned nerves regenerate at a rate of 2-3 mm/day, requiring approximately one year to heal^21^. Even with optimal medical and surgical management of peripheral nerve injury, muscle function is only partially restored^22^. This incomplete restoration is the failure of regenerating nerves to establish a proper muscle-nerve interface due to the disassembly of the neuromuscular junction (NMJ)^23^. The NMJ is comprised of three components including the terminal end of the motor axon, terminal Schwann cells and the end plate of muscle fibers containing acetylcholine receptors (AChR)^24^. By one week following peripheral nerve injury, AChR clusters disperse and AChR turnover shortens by a factor of ten leading to a marked decrease in the number of AChRs available for re-innervation^25^. Consequently, re-innervation may result in the recovery of only limited functionality.

Here we describe our efforts to engineer a device to prevent muscle atrophy and acetylcholine receptor degradation to improve subsequent re-innervation and decrease rehabilitation time. Three major approaches have been developed to better integrate electronics with the soft skeletal muscle tissue of the human body. First, gold wiring is printed onto a layer of PI, a flexible and biocompatible material, producing an elastic circuit board^26^. In addition to providing an electrical platform, the polyimide materials act as electrical insulation (**Figures 1E and 1G**). Second, two electrodes are rearranged in side-by-side parallel format, permitting conformity to curved surfaces^27^. Lastly, inspired by nature, circuit wiring was redesigned in fractal patterns that allow multidirectional stretching^18^.

## Materials and Methods

### MEA Fabrication

An outline of the device fabrication procedure is reported in **Figure S1**. The fabrication process begins with a clean 100 mm diameter silicon wafer (Test N-Type, <100>), which was used as the fabrication platform. 2) A 200 nm layer of polymethyl methacrylate (PMMA, 495 PMMA A6, Microchem) was spin coated onto the platform at 5,000 rpm for 60s. 3) A 10 μm layer of PI (PI 2574, HD Microsystems) was spin coated onto the platform at 2,700 rpm for 30 s and cured at 250° C for 2 h. 4) A 10 nm Titanium (Ti) adhesion layer and a 500 nm Gold (Au) layer was deposited onto the platform using the e-Beam deposition. 5) A 6 μm layer of Microposit s1813 photoresist was spin coated onto the platform at 1,500 rpm for 30 s and cured at 100° C for 3 h. 6) The Microposit s1813 photoresist was patterned via standard photolithography techniques using UV exposure. 7) The gold layer was etched using potassium iodide solution for 278s. The platform was dipped into the solution for 280 s inside an acid hood. The titanium adhesion layer was etched using 10:1:1 Deionized water/Hydrogen Fluoride/Hydrogen Peroxide solution for 10 s inside an acid hood. 8) A 10 μm PI encapsulation layer was spin coated at 2,700 rpm for 30 s and cured at 250° C for 2 h. 9) An 2 nm aluminum layer was deposited to protect the polyimide around the sites of the contact pads during reactive ion etching. 10) A 6 μm layer Microposit s1813 photoresist was spin coated at 5,000 rpm for 60 s and cured at 100° C for 3 h. 10) The Microposit s1813 photoresist was patterned via UV exposure. 11) The aluminum layer was etched at 50° C for 2 s in an acid hood. 12) The contacts were etched using reactive ion etch utilizing 100% oxygen. 12) The rest of the aluminum layer was etched at 50° C for 2s in an acid hood. 13) The PMMA base was dissolved using Acetone in and the device was detached from the platform. The electrodes were then cut into 1cm by 1cm squares.

### Surgical Implantation

All the animal experimental procedures were conducted in accordance with the guidelines of the Animal Care and Use Review Office (ACURO) of the US Army Medical Research and Materiel Command (USAMRMC) Office of Research Protections (ORP) and the Committee on Animal Care of Massachusetts Institute of Technology on female Lewis Rats (*n* = 10, age: 14 weeks). All devices were sterilized with ethylene oxide prior to implantation and allowed to degas for at least 24 h. Pre-emptive analgesia was administered subcutaneously at the following doses: buprenex (0.03 mg/kg) and meloxicam (1.0 mg/kg). All surgical procedures were carried out under isoflurane (2.0%)/oxygen inhalation. All surgeries were carried out in an aseptic field using aseptic technique. A 2 cm incision was made parallel to the femur. Blunt dissection through the vastus lateralis and biceps femoris muscle was performed to reveal sciatic nerve within the posterior fossa. The tibial nerve was identified as the largest and most central branch. This nerve was resected with Iris scissors as distally as possible (**Figure S8A**) and another sharp resection was made no less than 1.5 cm proximally. The 1.5-3 cm segment was removed from the posterior fossa (**Figure S8B**). The device was then secured to the gastrocnemius using four polyprolene sutures. The wires connected to the MEA were tunneled subcutaneously to reach the incision at the scalp. Headstages, connected to the end of the wire bundle, were mounted on the skull using dental ceramic. The animals were monitored until consciousness was regained. Post-operative checks were done 14 hours after surgery. Buprenex (0.03 mg/kg) was administered at 8-hour intervals for post-surgical pain management. Meloxicam (1.0 mg/kg) was administered every 24 hours for 48 hours. The animals were euthanized 14 days from the date of surgery.

### Histology

The tissue samples were harvested 14 days from the date of the surgical procedure. The samples were fixed in 4% formalin solution in phosphate buffered solution (PBS) for 24 hours. After, the samples were placed in 70% ethanol (with ultrapure Millipore water as diluent) and taken to the Koch Institute Histology Core where they were fixed in paraffin. Multiple axial slices, 5 µm in thickness, of the gastrocnemius muscles were taken from each of the paraffin blocks, fixed on microscope slides and stained with hemotoxylin & eosin (H&E), Mason's Trichrome Stain (MTS) and Pico Sirius Red (PSR) for analysis of muscle fiber area in addition to assessment of gross morphological changes and collagen deposition. Additional axial slices, 5 µm in thickness, from each paraffin block were fixed and stained with alpha bungarotoxin conjugated to Alexa-Flour 488 and then counterstained with DAPI.

### Morphometric Analysis of Muscle Fiber Area

From each sample stained with H&E, 200 muscle fibers were outlined from different locations along the gastrocnemius muscle using ImageJ software (**Figure S9A**). Using the “measure” function, 50 muscle fibers were outlined among 4 different fields of view using an EVOS Digital Microscope on brightfield at 40 times magnification and obtained a cross sectional area. Areas are reported in Arbitrary Units (AU).

### Immunofluorescence Morphometric Analysis

The images were collected using a DeltaVision fluorescent microscope and analyzed using ImageJ software (**Figure S10B**). Using the split channels feature, each individual composite image was separated into the three RGB channels. The green channel, with the acetylcholine receptor stain, was put under threshold at below 0.39% to rid the image of background autofluorescence and keep only positive signal. This threshold remained the same for all the images for consistency. The residual signal after threshold cutoff was then measured using the analyze particles feature. The size and circularity were constant, above 5 pixels and between 0.00-1.00 respectively. Outlines were overlaid onto the positive signal and the total area was measured. This macro was run on 10 different fields of view at 40 times magnification and the output area of the signal was labelled and measured. This was repeated for both the groups in the study. Areas are reported in Arbitrary Units (AU).

### In Vivo Functional Electrical Stimulation

**Figure 2:**
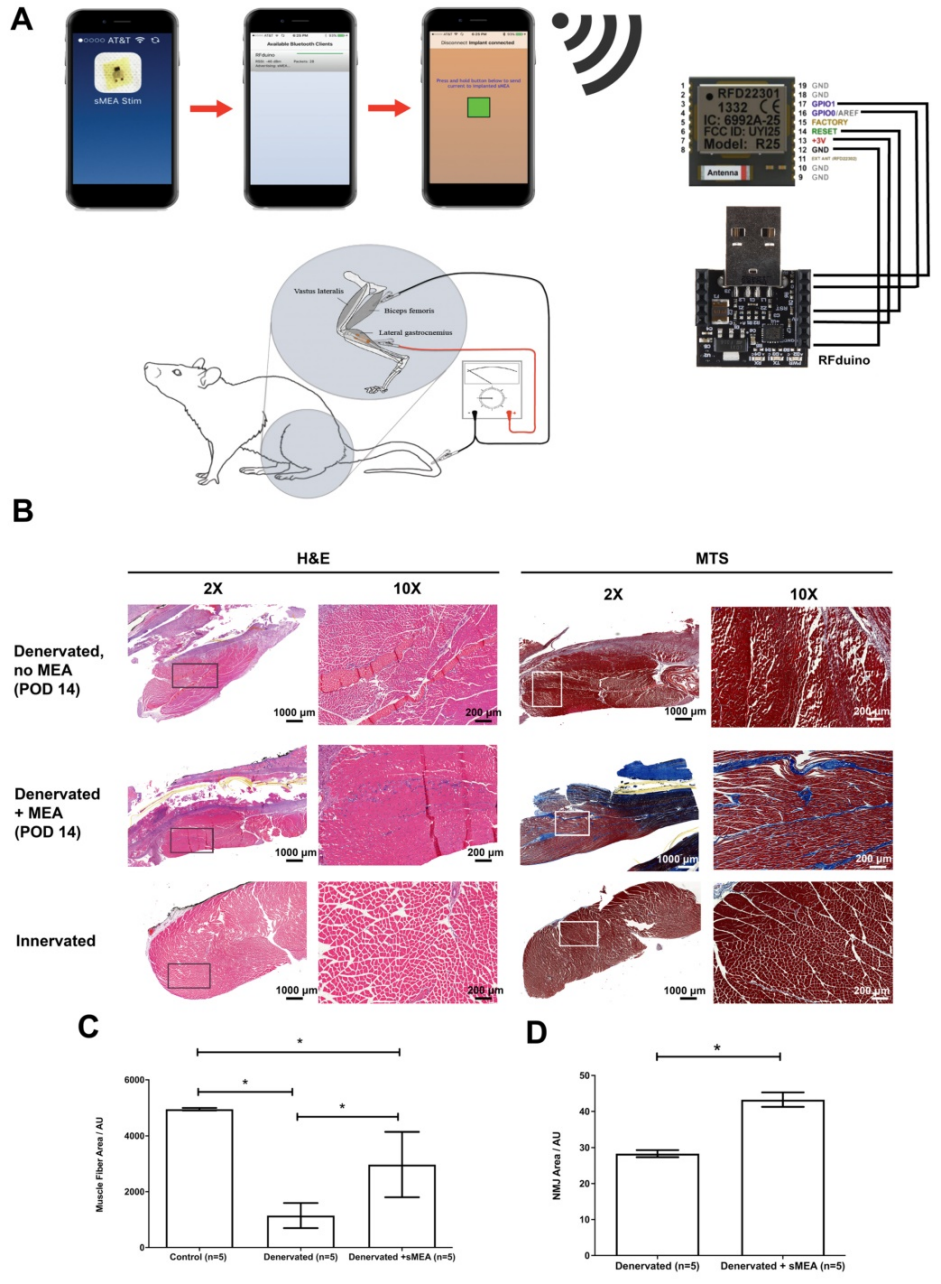
(A) The duration, voltage and frequency of stimulation are controlled by a phone application and an rFDuino microcontroller with a Bluetooth client. A total of 10 animals underwent tibial nerve injury surgeries of which 5 animals were implanted with electrodes. After post-operative day 14 (POD 14), the gastrocnemius muscles were harvested for histological and immunohistochemical staining. (B) Cross-section at 2X and 10X magnification of H&E and Mason's Trichrome (MTS) stained muscle fibers from: (1) nstimulated, denervated muscle (top, “Denervated, no MEA”), (2) muscle fibers from MEA-stimulated denervated muscles (middle, “Denervated + MEA”) and (3) muscle fibers from unstimulated, innervated muscle (bottom, “Control”). Scale bars = 1000 μm for 2X magnification and 200 μm for 10X magnification (C) Measurement of muscle fiber cross sectional areas comparing “Control” (left), “Denervated” (no MEA, middle) and “Denervated + MEA” (right). Error bars are ± S.E.M. and * denotes p < 0.05 by Kruskal-Wallis test with Dunn's Multiple Comparison Post Test. For the control (innervated gastrocnemius muscle) cases, 1600 fibers were measured from 5 animals. For the 5 animals with denervated gastrocnemius muscles without MEA stimulation, 1600 fibers were measured from 8 animals. For the MEA case, 955 fibers were measured from 5 animals. Muscle fiber areas are reported in Arbitrary Units (AU). (D) Mean AChR area for non-stimulated and stimulated muscles. There was significantly more area in the stimulation case. Error bars are ± S.E.M. and * denotes p < 0.05 by Mann Whitney test. Among the 5 animals with denervated gastrocnemius muscles without stimulation, 3160 readings were taken. Among the 5 animals with denervated gastrocnemius muscles with MEA stimulation, 2697 readings were taken. Areas are reported in Arbitrary Units (AU).

Electrical input leads to and output leads from the MEA travel through the rFDuino microcontroller with an on-board wireless Bluetooth client (**Figure 2a**). An iPhone application was created to control amplitude, frequency and interval lengths through the Arduino Integrated Development Environment (IDE) code. Using the Arduino IDE, we were able to pre-program the desired parameters for functional electrical stimulation and upload it to the rfDuino microcontroller. As the rfDuino is powered by a small low power coin battery (CR2032 Lithium metal 3V 250mAh button cell battery), it starts the BLE stack. Using XCode, we use a system to pair an iPhone to the rfDuino via Bluetooth; one paired the phone acted as a controller for the pre-programmed parameters given to the microcontroller. Upon the input of a button press on the phone, the binary input data was sent wirelessly to the microcontroller which began the stimulation procedure with the desired parameters. The system drew the necessary current from the low-power coin battery at the set programmed intervals and outputted it to the MEA. The rfDuino was wired to the MEA using thin medical grade stranded stainless steel wire (CoonerWire AS633). The current travelled through the MEA and out of the small contact pads where it interfaced with the tissue.

### In Vivo Epimysial Recording

One Lewis rat was anesthetized by isoflurane (2%)/oxygen inhalation. An opening incision was made halfway along the length of the femur. Blunt dissection was performed at the fat pad between the vastus lateralis/biceps femoris muscle plains to reveal the sciatic nerve. The sciatic nerve was then isolated and lifted 2 cm using a hooked nerve stimulator to avoid muscle contact.

**Figure 3:**
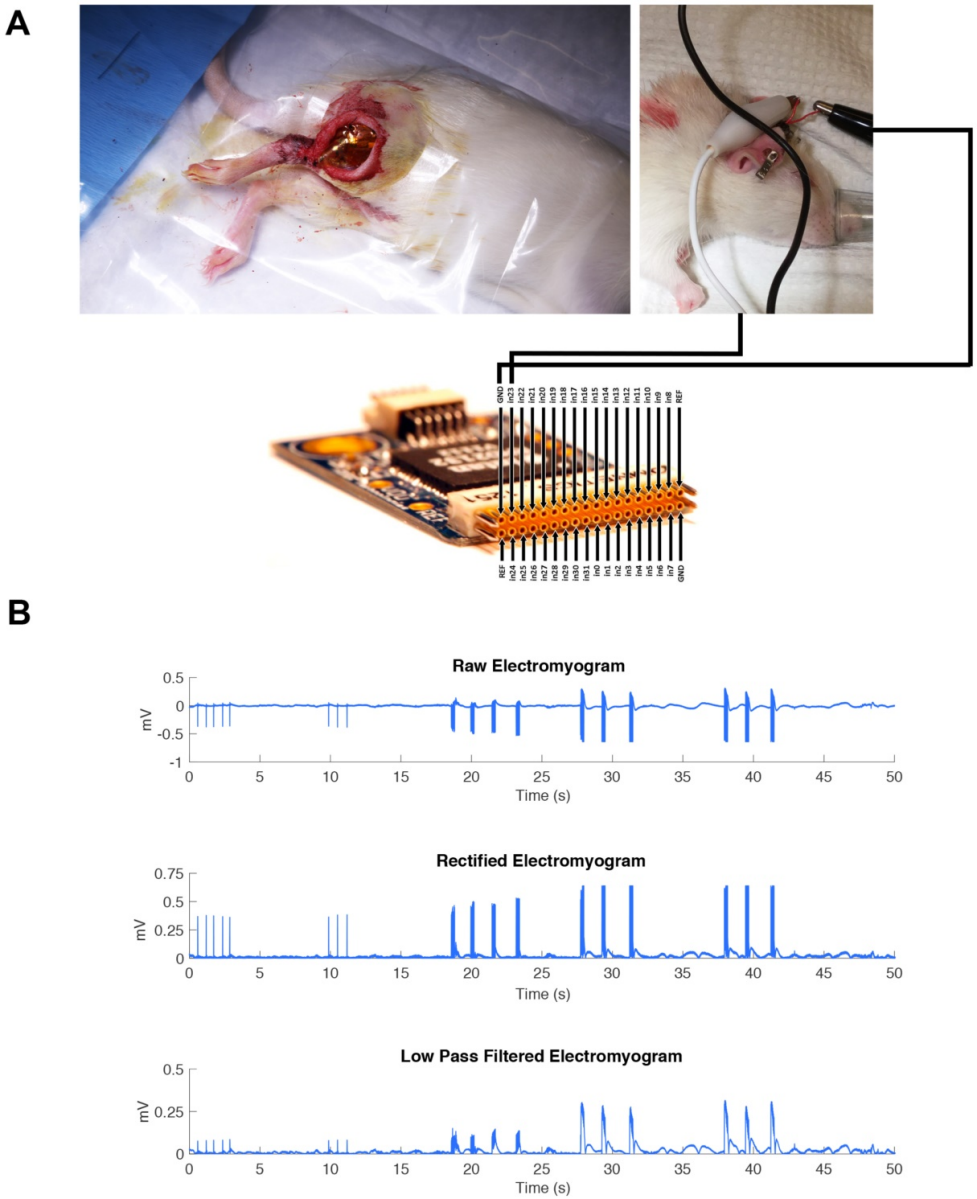
(A) The MEA device was connected to an RHD2000-Series Amplifier Evaluation System (Intan Technologies). The RHD2000 chip output the EMG signal to a computer where the data was processed using MATLAB. The MEA was placed flat on top of the biceps femoris surface for ideal muscle contact away from the sciatic nerve. A ground electrode was inserted intramuscularly to provide a pathway for electrical conductivity.” (B) A representative EMG is shown immediately after electrode implantation in one animal, including all post-processing steps, to demonstrate the recording capability of the MEA during direct stimulation of the sciatic nerve in trains of three pulsatile stimulations.

The hooked nerve electrode was attached to the Model S88X Grass Stimulator (Astro-Med Inc.). The Grass stimulator was set to output a biphasic train of 400 ms with a pulse width of 200 µs per phase. An amplitude sweep was used to determine if the MEA would be able to pick up changes in electrical signal by increasing the amplitude of signal for each pulse train. The recording was performed on three consecutive pulse trains. The controlled electrical current from the Grass stimulator flowed to the hooked nerve electrode to stimulate the isolated sciatic nerve. The MEA device was connected to an RHD2000-Series Amplifier Evaluation System (Intan Technologies) which allows for recording biopotential signals from up to 256 low-noise amplifier channels (**Figure 3A**). The MEA was placed flat on the dorsal surface of the biceps femoris surface for ideal muscle contact away from the sciatic nerve. In addition, a ground electrode was inserted subcutaneously to provide a pathway for electrical conductivity.

Controlled electrical current with varied stimulation parameters was then sent through the nerve stimulator to the isolated sciatic nerve which resulted in observable muscle contractions. The epimysial device took in the EMG activity of these muscle contractions and sent the recording data to the computer. The data was further amplified, rectified, and low-pass filtered in MATLAB.

### Statistical Analysis

No pre-processing of the H&E images or the microscopy images was performed. The data is presented as standard error of measurement (S.E.M.) and number of measurements shown in **Tables S1 and S2**.Q-Q plots of all muscle fiber cross sectional area data (**Figure S3** and **S4**) and acetylcholine receptor area data (**Figure S5 and S6**) indicated a non-normal distribution. This non-Gaussian distribution was further confirmed by the Shapiro-Wilk test. The lack of normality precluded the use of parametric statistical analyses (i.e. Student's t-test and ANOVA). To compare 2 means, the non-parametric Mann Whitney test for independent groups was used. To compare more than 2 independent groups, the non-parametric Kruskal-Wallis test with Dunn's Multiple Comparison Post Test were performed. Differences were considered statistically significant if p < 0.05. Statistical tests were performed using GraphPad Prism Version 5.00 and R Version 3.4.3.

## Results

### Mechanical Testing of the Flexible Multi-Electrode Array (MEA)

Mechanical testing of the MEA was also performed in order to determine its elastic properties under simulated *in vivo* stresses. The modulus match between the MEA and underlying muscle and tendon tissue is important for prevention of many acute and chronic problems stemming from implantation trauma, micromotion damage and inflammation at the implantation site.

As reported in **Figure S2**, the MEA is able to withstand up to 5.5 N of load before failure at 20 percent elongation. The Young's modulus (E) at maximum elongation before the circuit cracked was 1.67 GPa. The Young's modulus for tendon is approximately 1 GPa and the Young's modulus for a passive muscle being stretched is 10 kPa(*28*). After maximal flexion using uniaxial load strain, the MEA was able to still conduct electricity and no evidence of metal cracking was found after viewing the gold traces through Scanning Electronic Microscopy (SEM) (**Figure 1F**).

### Therapeutic Efficacy

We determined the therapeutic effects of muscle stimulation using the MEA device to prevent atrophy of denervated muscle and degradation of the acetylcholine receptors. Five MEA devices were implanted along the surface of the gastrocnemius muscles of five Lewis rats after critical 1.5-3 cm segment resection of the tibial nerves. The five animals were wirelessly stimulated with 500 mV, 2 Hz of FES for an hour a day, five days a week for 14 days (**Figure 2A**). The animals were euthanized and the muscles were collected 14 days after surgery. **Figures 2B, 2C** and **Table S1** show the treatment results on muscle fiber cross-sectional area. Three conditions were quantified: 1) no FES using the MEA (“Denervated”); 2) denervated muscle with FES using the MEA (“Denervated + MEA”); and 3) intact nerve with no FES (“Control”). Hemotoxylin & eosin (H&E) and Mason's Trichome Stain (MTS) were used to stain the muscle fibers. Though still lower than Wild Type controls, the MEA-treated denervated muscles did show a marked improvement in muscle fiber cross sectional area over non-stimulated denervated controls. The muscle fiber areas from animals treated with the MEA-mediated FES show a 160% increase in average area compared with untreated, denervated animals (p < 0.05).

We also tested whether MEA-mediated FES led to stabilization of acetylcholine receptors which would lead to better re-innervation. The muscles were analyzed with immunohistochemistry (**Table S2**) using α-bungarotoxin (α-BTX) to label the post-synaptic nicotinic acetylcholine receptors (nAChRs). There was a significant (p < 0.05) increase in the area of α-BTX stained particles among the MEA-mediated FES treated group compared to the untreated (“Denervated”) group (**Figure 2D**).

### Epimysial Recording

For a complete system, the MEA must also monitor recovery of the subject by producing an electromyogram (EMG). Epimysial electrodes must monitor muscle activity to determine appropriate therapy. We sought to study the recording capability of the MEA to generate a simple EMG at different amplitudes of neural stimulation.

The sciatic nerve was stimulated in one animal model at increasing amplitudes: 20 mA, 40 mA, and 60 mA as a pulse train with a pulse width of 200 µs. The MEA was implanted on the dorsal surface of the gastrocnemius muscle to monitor the muscle contractions. (**Figure 3A**). As reported in **Figure 3B**, we were able to identify clearly each of the three major pulse trains sent out and determine the difference in amplitude of the signal. The recording trace shows the MEA was able to record these electrical signals well *in vivo*.

## Discussion

Functional electrical stimulation (FES), which substitutes neuromotor control by electrical stimulation, is used clinically to treat muscle atrophy^28^, ^29^. Through excitation-contraction coupling, a daily stimulation regimen upregulates the protein synthesis pathway in muscle fibers, downregulates the protein degradation pathway, and affects extracellular matrix remodeling in the muscle^30^. Additionally, FES significantly increases the number and size of AChR clusters available for NMJ formation^31^. Intramuscular electrodes have generally failed in the clinical setting due to destruction of the interfacing tissue by inflammatory responses^29^. Transcutaneous electrodes eliminate the damage associated with electrode penetration, but require high current levels and may result in unwanted nerve excitation^32^. Thus, no effective solution currently exists for treating muscle denervation atrophy during the course of nerve repair. Furthermore, additional information is needed with regard to the kinetics of nerve regeneration, motor re-innervation, and the optimal patterns of electrical stimulation^33^. Hence, there remains a need for an effective, continuous interface that improves stimulation of denervated muscle without tissue destruction and allows for real-time *in vivo* monitoring of nerve regeneration.

The multielectrode array reported here integrates the flexibility of a polymer-based electrode with the stretchability of fractal patterned metal electrodes. The elastic properties of the arrays are in range of native muscles and tendons, ensuring better mechanical compliance. This foundational work demonstrated the feasibility the MEA fabrication, the successful prevention of muscle atrophy and an attenuation of the loss of acetylcholine receptors after peripheral nerve injury.

Towards achieving dual-use capabilities, the MEA function was further expanded by its ability to record epimysial activity. By combining the therapeutic benefits with wireless control and telemetry, this technology may allow for the creation of patient-specific treatments with real-time updating. Understanding that a comprehensive critical peripheral nerve injury therapy must allow for axonal regeneration across the length of the nerve defect to reach the muscle and reinnervate, the MEA's benefits of denervated muscle and acetylcholine receptor maintenance may also be combined with additional techniques including engineered nerve constructs^34^ or nerve conduits^35^. This combined approach may increase the number of axons that successfully regenerate to end organs and, as a consequence, promote nerve regeneration and motor and sensory functional recovery after nerve injury.

There are a few limitations of this study. In this proof-of-concept study, the outcomes and recording capability of this electrode are not compared with classic (non-flexible) epimysial electrodes. One future study will involve direct comparison of Young's modulus, impedance after tension loading and *in vivo* recording capabilities as well as genetic and histological markers of foreign body associated inflammation with an electrode fabricated without the Peano curve motifs as well as in comparison with more rigid electrodes currently used clinically such as the Livermore^36^, ^37^, Utah^38^, ^39^ and Michigan^40^-^42^ electrode arrays. Future work will focus on biocompatibility and prevention of fibrotic capsule formation to improve both therapeutic efficacy and accuracy of epimysial recording. Towards clinical translation, a tolerability and safety study investigating long-term application both in rats and in other animal models including pigs will be conducted.

## Conclusions

Using inspiration from the semiconductor fabrication industry, we have developed a flexible electrode array for epimysial implantation. In contrast to previously described electrodes, this proof-of-concept multielectrode array reported here integrates the flexibility of a polymer-based electrode with fractal patterned metal traces. To help prevent against fibrosis-related issues, biocompatible materials like polyimide and gold were utilized in fabrication. The elastic properties of the arrays are in range of native muscles and tendons, ensuring better mechanical compliance. Our primary functional goal was to preserve muscle strength during nerve regeneration to allow for quicker rehabilitation from injury. In a rodent peripheral nerve injury model, the arrays successfully diminished muscle atrophy and attenuated the loss of neuromuscular junctions during peripheral nerve injury. Muscle fiber and neuromuscular junction area showed a marked increase in denervated animals with MEA-mediated FES compared to untreated, denervated animals. In addition, the electrode's functionality was further expanded by its ability to record epimysial electrical activity. EMG recordings from the MEA picked up strength and frequency of gastrocnemius muscle contraction based on stimulation protocols. From our findings, both of the therapeutic and diagnostic abilities of this device showed promise *in vivo*. With this dual-functioning electrode, we can now apply therapeutic electrical stimulation regimens and also monitor the recovery process in real-time.
