# Supplementary material for: Flexible Multielectrode Array for Skeletal Muscle Conditioning, Acetylcholine Receptor Stabilization and Epimysial Recording After Critical Peripheral Nerve Injury
Source: Theranostics. 2019 Sep 21;9(23):7099–107. doi: 10.7150/thno.35436 (PMC6815960; doi:10.7150/thno.35436)
Supplement: Supplementary file 1 — Supplementary figures and tables. [file thnov09p7099s1.pdf]

Supplemental Material

Flexible Multielectrode Array for Skeletal Muscle Conditioning, Acetylcholine Receptor Stabilization and Epimysial Recording After Critical Peripheral Nerve Injury

*Malia McAvoy**, Jonathan K. Tsosie**, Keval N. Vyas**, Omar F. Khan, Kaitlyn Sadtler, Robert Langer, Daniel G. Anderson*[†]
**These authors contributed equally to this work.

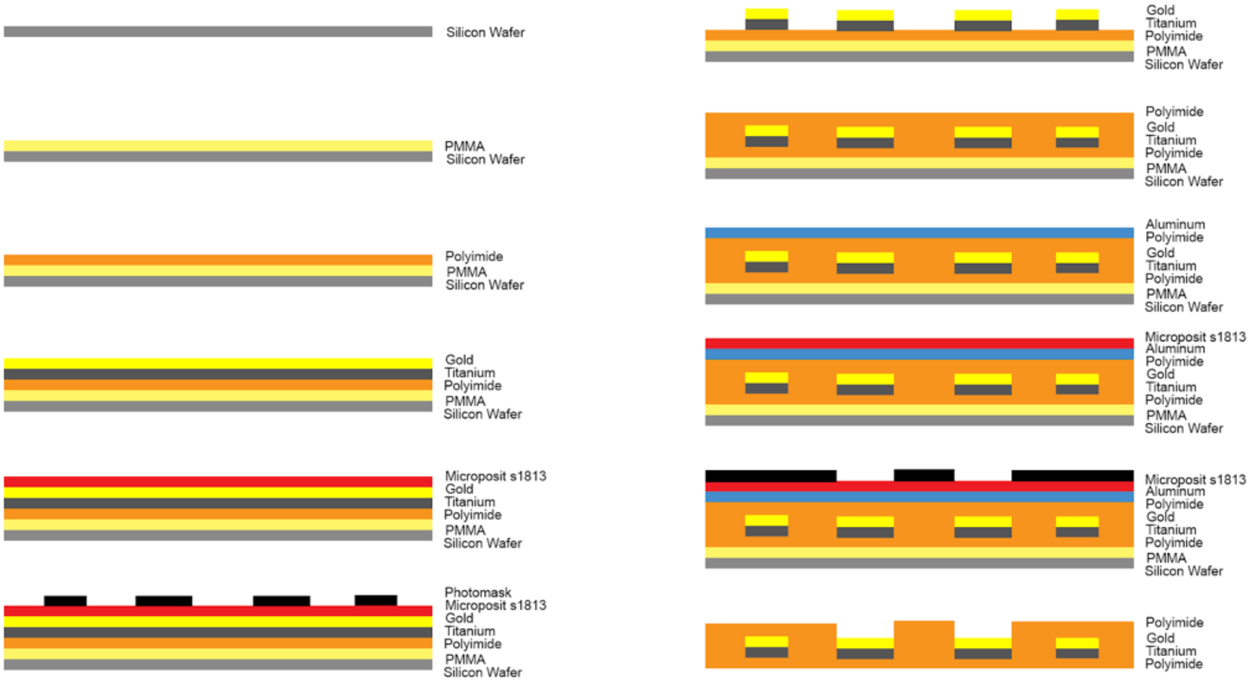

Figure S 1. Electrode fabrication protocol.

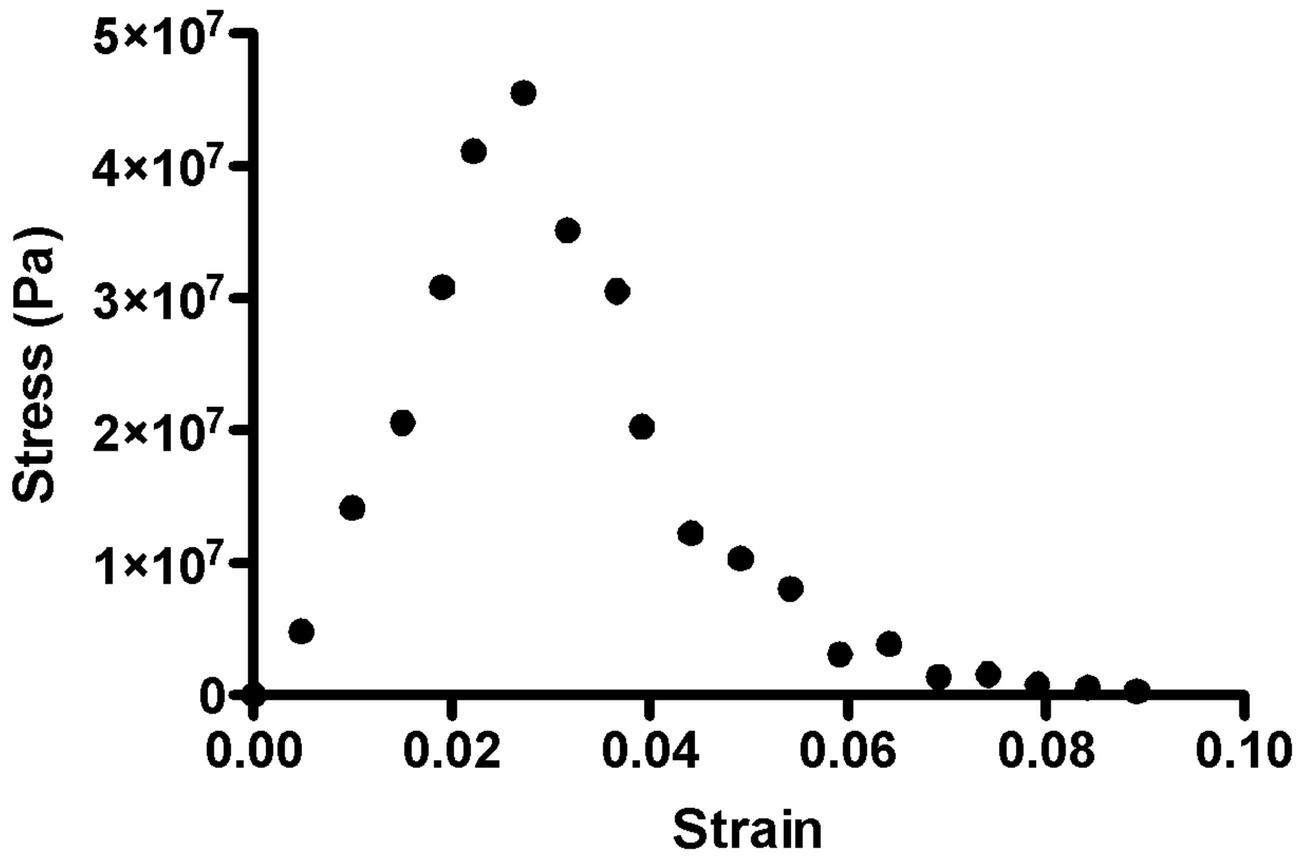

Figure S 2. Tensile strength of the MEA. A maximum load of 5.5 N was achieved at an extension of 0.25 mm. The tensile strength was 4.48 x 10^7 Pa and the Young's modulus was 1.7 x 10^9 Pa.

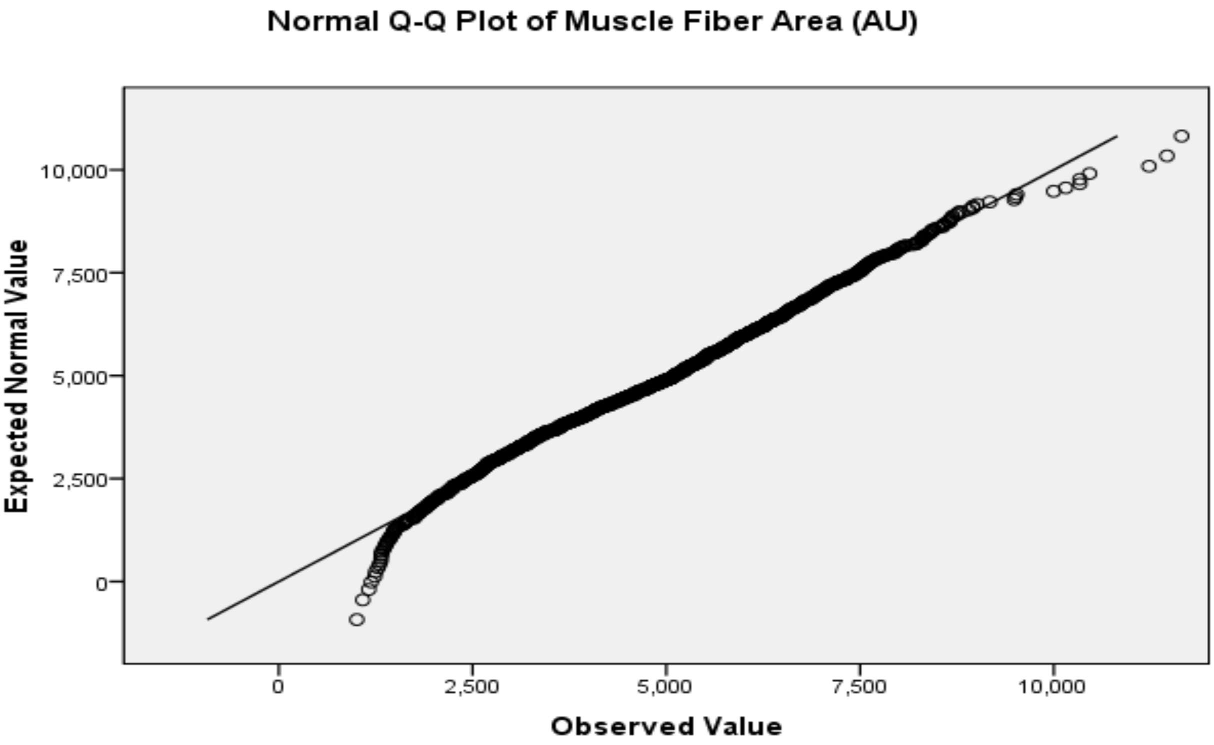

Figure S 3. Q-Q plot of Control muscle fiber area data.

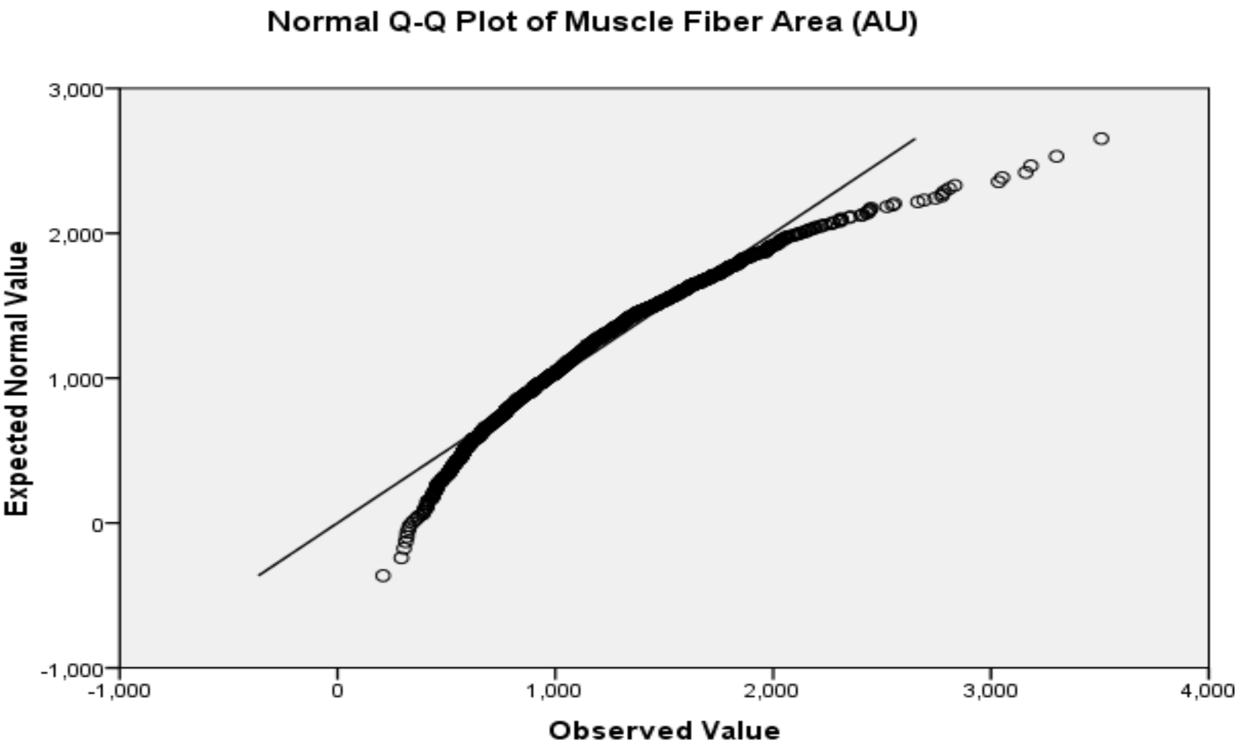

Figure S 4. Q-Q plot of Denervated (not stimulated) muscle fiber area data.

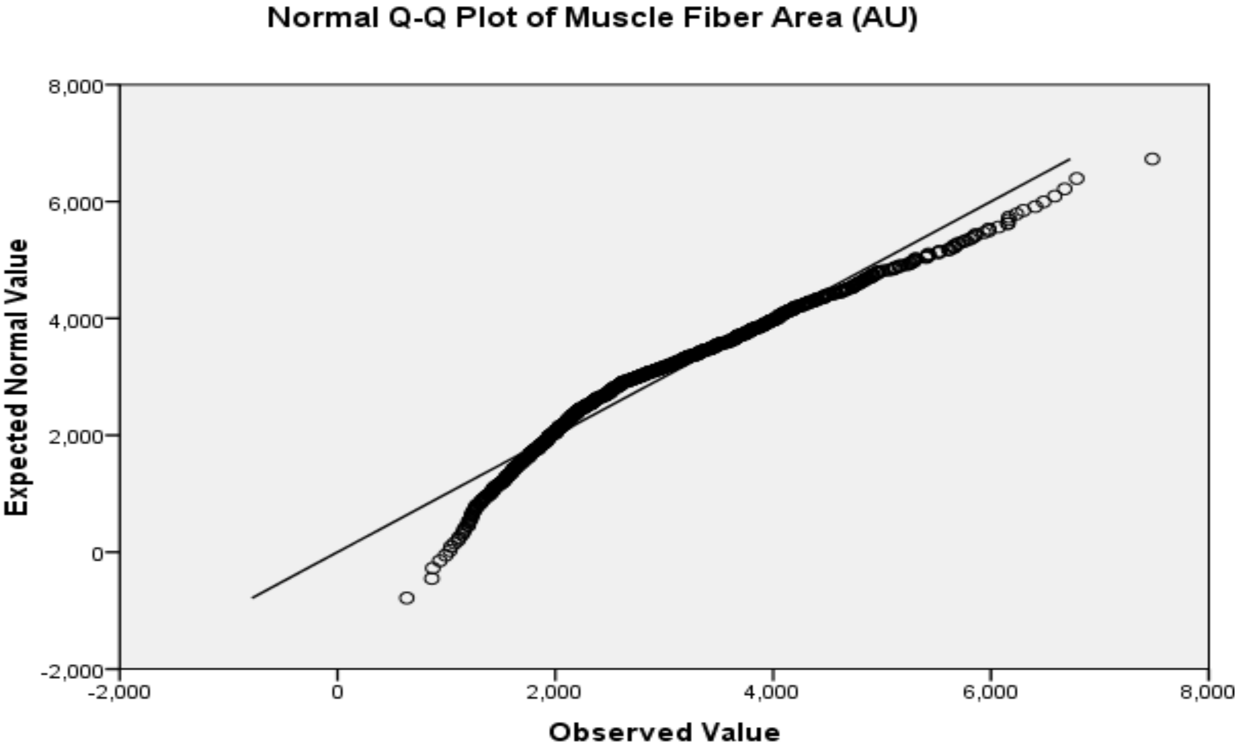

Figure S 5. Q-Q plot of Denervated + MEA (stimulated) muscle fiber area data.

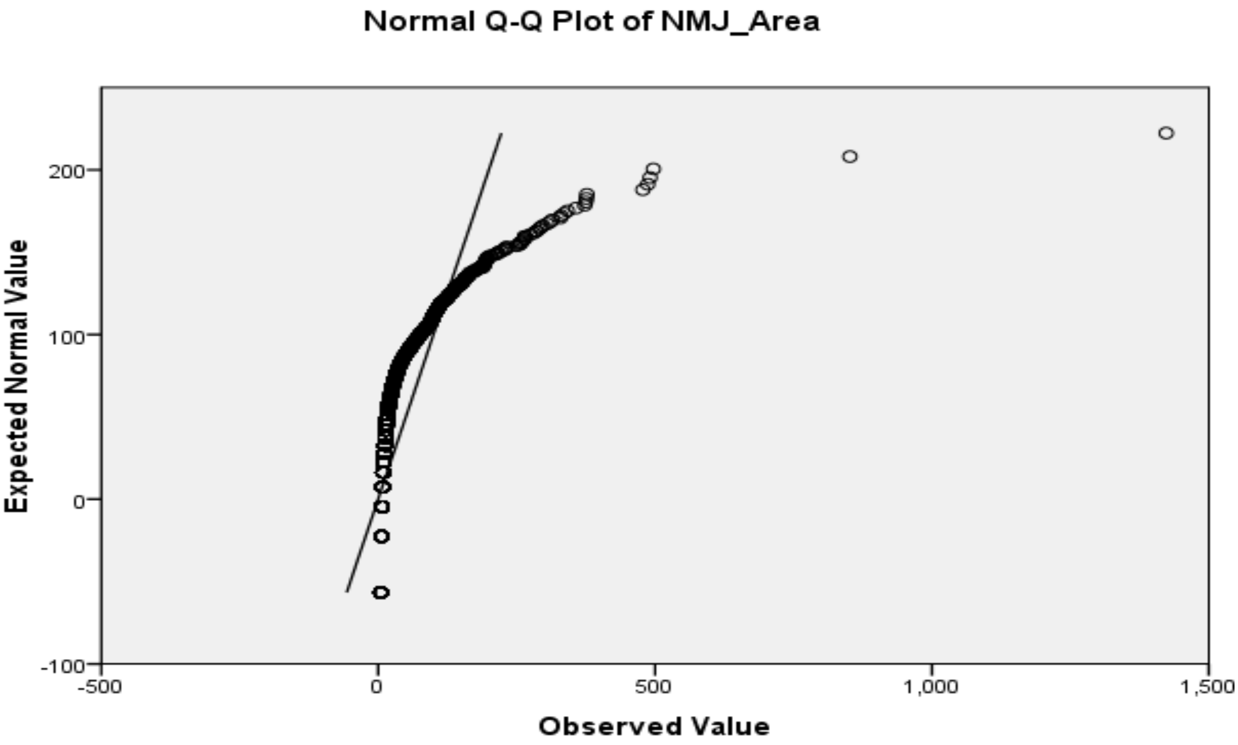

Figure S 6. Q-Q plot of acetylcholine receptor area data for Denervated (not stimulated) muscle.

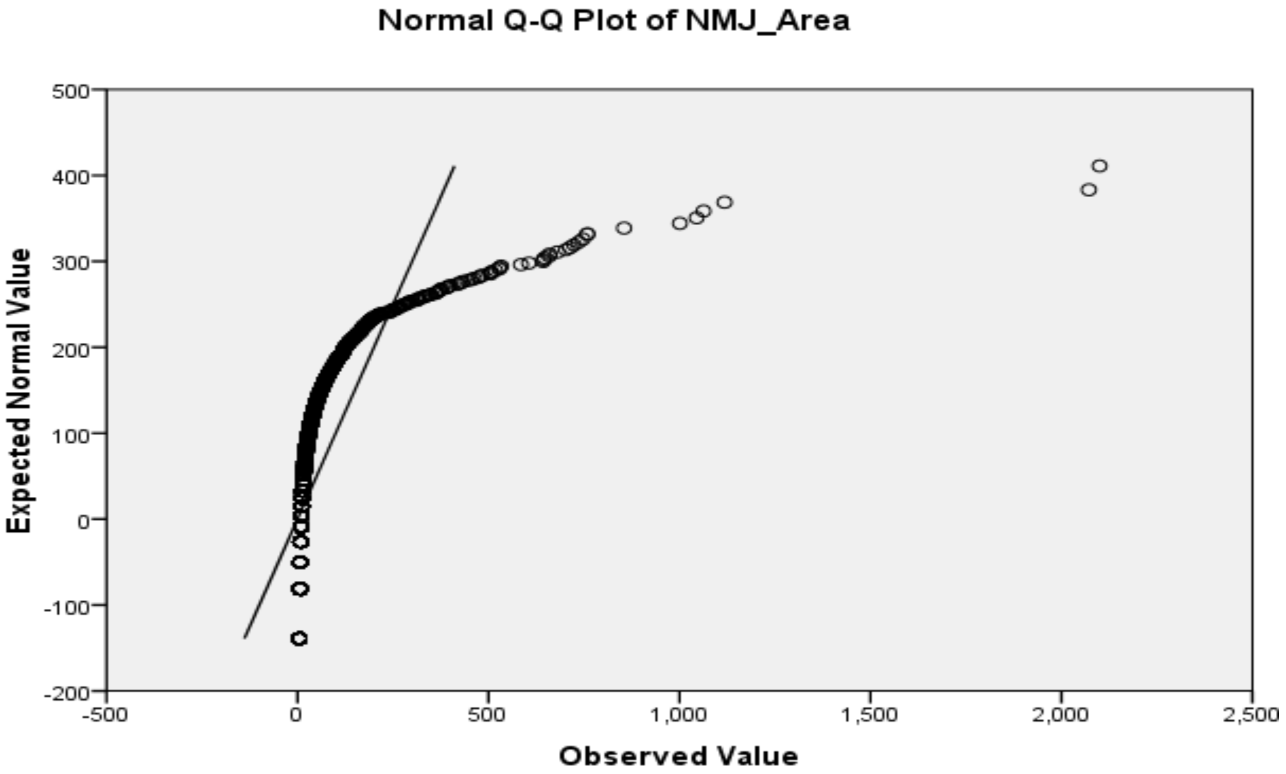

Figure S 7. Q-Q plot of acetylcholine receptor area data for Denervated + MEA (stimulated) muscle.

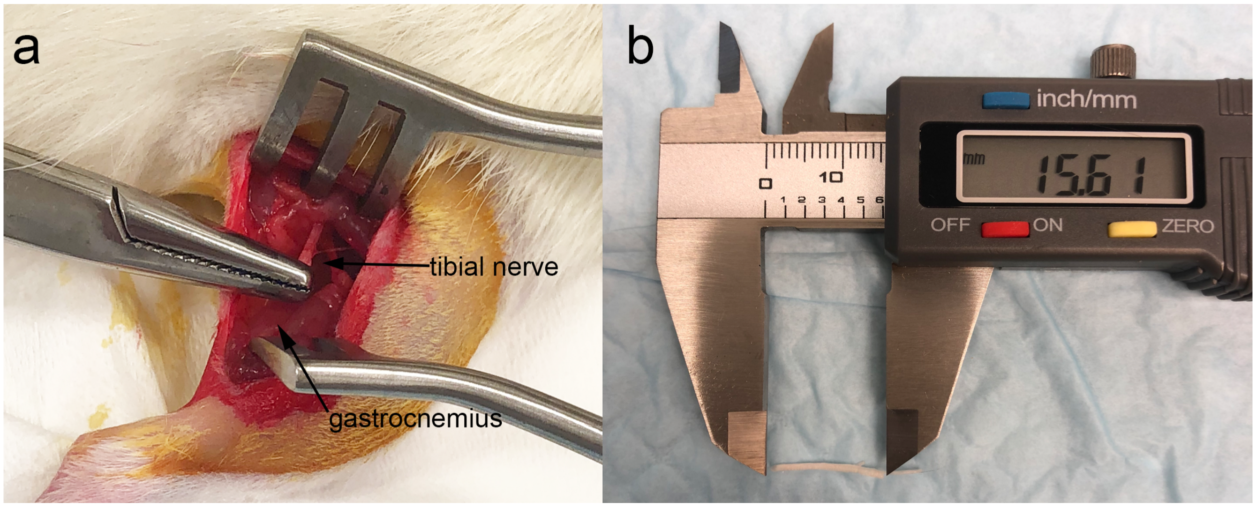

Figure S 8. The critical peripheral nerve injury model consisted of removing a 1.5-3 cm segment of the distal tibial nerve. (a) The tibial nerve is identified and the distal end (held by clamp) is cut. (b) A proximal cut is also made to remove a 1.5 cm nerve segment.

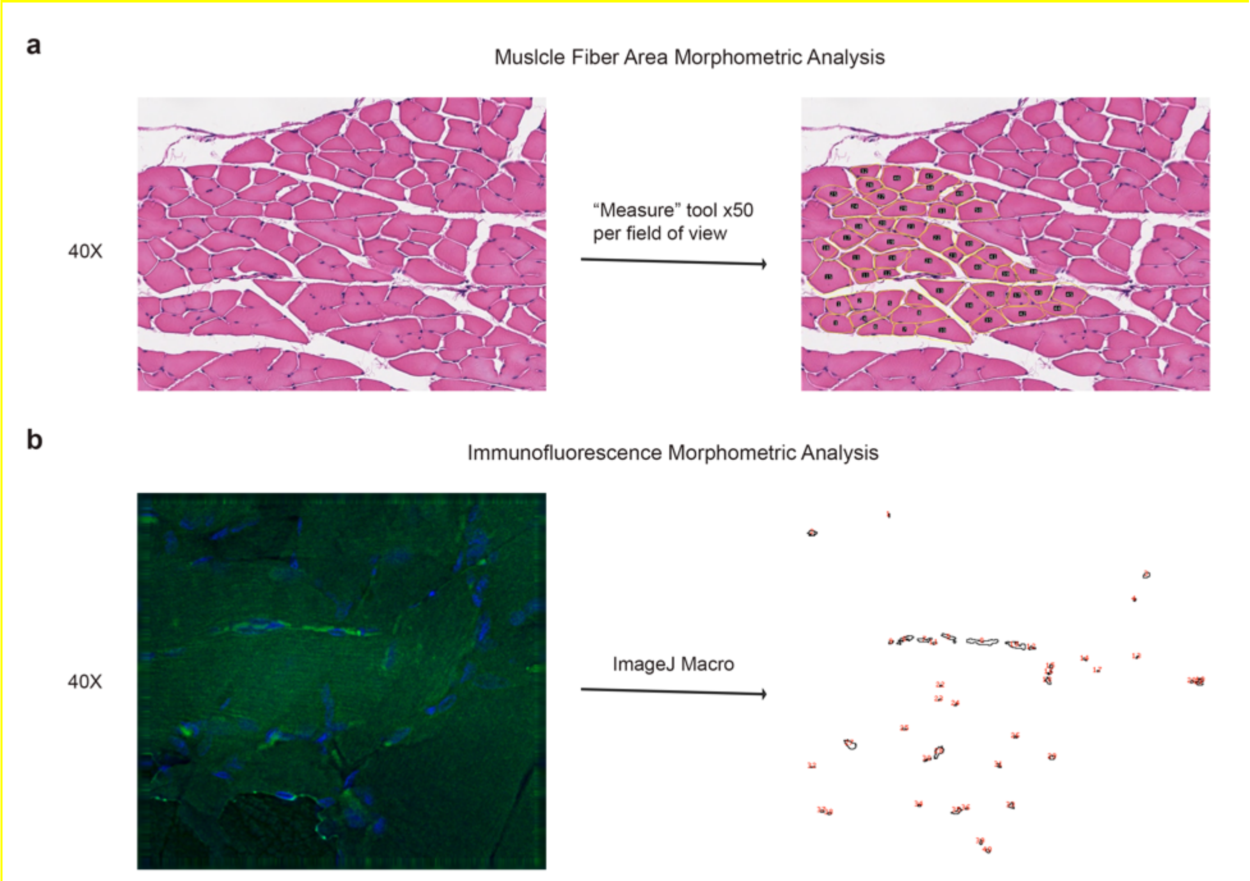

Figure S 10. (a) The muscle fiber area was calculated using the ImageJ “measure” tool for 50 muscle fibers per field of view at 40 times magnification under bright field microscopy. There were 4 total fields of view analyzed. (b) The acetylcholine receptor area indicated by alpha-bungarotoxin immunofluorescence staining (green) was measured using an ImageJ macro on images collected at 40 times magnification viewed using confocal microscopy. There were 10 total fields of view analyzed.

	Control, Intact nerve	Denervated, no FES	Denervated + MEA, with FES
Mean Muscle Fiber Cross-Sectional Area (AU)	4948	1145	2971
S.E.M.	43.69	11.23	37.82
n	1600	1600	955

Table S 1. Descriptive statistics for muscle fiber cross-sectional areas in rat in vivo experiments. Areas are reported in Arbitrary Units (AU). The standard error of measurement (S.E.M.) and the number of measurements (n) are also shown. FES = Functional Electrical Stimulation.

	Denervated, no FES	Denervated + MEA, with FES
Mean Neuromuscular Junction Area (AU)	28.33	43.28
S.E.M.	0.9740	2.022
n	3160	2697

Table S 2. Descriptive statistics for acetylcholine receptor areas in rat *in vivo* experiments. Areas are reported in Arbitrary Units (AU). The standard error of measurement (S.E.M.) and the number of measurements (n) are also shown. FES = Functional Electrical Stimulation.
